# 3D printing in clinical podiatry: a pilot study and review

**DOI:** 10.1186/1757-1146-8-S2-O41

**Published:** 2015-09-22

**Authors:** Cylie Williams, Alicia James, Michael P Chae, David J Hunter-Smith

**Affiliations:** 1 Peninsula Health, Community Health, Frankston, VIC, 3199, Australia; 2 Monash University, Department of Physiotherapy, VIC, 3199, Australia; 3 Peninsula Health, High Risk Foot Service, Frankston, VIC 3199, Australia; 4 Peninsula Health, Department of Surgery, Frankston, VIC, 3199, Australia; 5 Monash University, Department of Medicine, Clayton, VIC, 3199, Australia

**Keywords:** 3D printing, new technology, footwear

## Background

Advanced and new technologies within the health sector are aimed to increase efficacy and reduce cost. As a profession, podiatry has seen exponential growth in the use of 3D (three-dimensional) scanning in the clinical domain. It has been used within orthotic manufacture to improve timely responses in both production and rapid dispensing. It has also been used within research to better understand foot structure and function.

3D printing is the next technological step, which will impact the podiatric management of common disorders. This presentation explores its potential uses and describes a new method of prosthetic construction based on 3D printing methodology.

## Process

The use of 3D printing technology for pre-operative planning is well established at Peninsula Health across multiple surgical disciplines. The 3D-printed models generated from CT (computed tomography) and MRI (magnetic resonance imaging) scans are currently undergoing validation clinical trials. The combination of 2D imaging and 3D haptic models can be useful in podiatry, specifically in the production of custom-made shoe fillers for people who have undergone transmetatarsal amputation or toe amputation. This process is currently being studied within Peninsula Health's High Risk Foot Clinic with the support of Department of Surgery.

## Findings

The use of a shoe filler to improve shoe fitting is a common practice following amputation, however it is associated with a high cost ($500-$800 per patient). We describe the use of a 3D rendered CT image of the foot within the shoe that enables a 3D printed shoe filler to be constructed at an estimated cost of $30. Our assessment of the practical use of these shoe fillers, durability and patient acceptance will be presented.

## Conclusion

3D printing has a role in podiatry however the application, its advantages and disadvantages are yet to be fully explored. This new and innovative technology introduces exciting future opportunities in the clinical setting.

